# Exogenous 8-Hydroxydeoxyguanosine Attenuates PM_2.5_-Induced Inflammation in Human Bronchial Epithelial Cells by Decreasing NLRP3 Inflammasome Activation

**DOI:** 10.3390/antiox12061189

**Published:** 2023-05-31

**Authors:** Jihye Bang, Kuk Hui Son, Hye-Ryeon Heo, Eunsook Park, Hyun-Jeong Kwak, Kyung-Ok Uhm, Myung-Hee Chung, Young-Youl Kim, Hyun Joung Lim

**Affiliations:** 1 Division of Allergy and Respiratory Disease Research, Department of Chronic Disease Convergence Research, National Institute of Health, Osong Health Technology Administration Complex 187, Osongsaengmyeong 2-ro, Osong-eup, Heungdeok-gu, Cheongju-si 28159, Republic of Korea; bjh0920@korea.kr (J.B.); heohr@korea.kr (H.-R.H.); eunsookpark@korea.kr (E.P.); uhm12345@korea.kr (K.-O.U.); youngyk07@korea.kr (Y.-Y.K.); 2 Gachon University Gil Medical Center, Department of Thoracic and Cardiovascular Surgery, College of Medicine, Gachon University, 21, Namdong-daero 774 beon-gil, Namdong-gu, Incheon 21565, Republic of Korea; dr632@gilhospital.com; 3 Major of Life Science, Division of Bioconvergence, College of Convergence and Integrated Science, Kyonggi University, 154-42 Gwanggosan-ro, Yeongtong-gu, Suwon-si 16227, Republic of Korea; hjkwak@kyonggi.ac.kr; 4 Lee Gil Ya Cancer and Diabetes Institute, Gachon University, 155, Gaetbeol-ro, Yeonsu-ku, Incheon 21999, Republic of Korea; mhchung@snu.ac.kr

**Keywords:** particulate matter 2.5, lung injury, reactive oxygen species, 8-hydroxydeoxyguanosine, NLRP3 inflammasome

## Abstract

Particulate matter 2.5 (PM_2.5_) induces lung injury by increasing the generation of reactive oxygen species (ROS) and inflammation. ROS aggravates NLRP3 inflammasome activation, which activates caspase-1, IL-1β, and IL-18 and induces pyroptosis; these factors propagate inflammation. In contrast, treatment with exogenous 8-hydroxydeoxyguanosine (8-OHdG) decreases RAC1 activity and eventually decreases dinucleotide phosphate oxidase (NOX) and ROS generation. To establish modalities that would mitigate PM_2.5_-induced lung injury, we evaluated whether 8-OHdG decreased PM_2.5_-induced ROS generation and NLRP3 inflammasome activation in BEAS-2B cells. CCK-8 and lactate dehydrogenase assays were used to determine the treatment concentration. Fluorescence intensity, Western blotting, enzyme-linked immunosorbent assay, and immunoblotting assays were also performed. Treatment with 80 μg/mL PM_2.5_ increased ROS generation, RAC1 activity, NOX1 expression, NLRP3 inflammasome (NLRP3, ASC, and caspase-1) activity, and IL-1β and IL-18 levels in cells; treatment with 10 μg/mL 8-OHdG significantly attenuated these effects. Furthermore, similar results, such as reduced expression of NOX1, NLRP3, ASC, and caspase-1, were observed in PM_2.5_-treated BEAS-2B cells when treated with an RAC1 inhibitor. These results show that 8-OHdG mitigates ROS generation and NLRP3 inflammation by inhibiting RAC1 activity and NOX1 expression in respiratory cells exposed to PM_2.5_.

## 1. Introduction

Particulate matter 2.5 (PM_2.5_) has a small diameter (<2.5 μm) and large surface area [1,2]. Due to these characteristics, PM_2.5_ has increased stagnation time and propagation distance, which causes it to easily infiltrate terminal airways such as the alveoli and readily dissolve into the circulatory system from the respiratory tract [1,2]. Since PM_2.5_ also acts as a carrier of various materials such as nitrate, carbon particles, ammonium salt, bacteria, and viruses [3], PM_2.5_ induces the secretion of various inflammatory cytokines such as tumor necrosis factor (TNF)-α, interleukin (IL)-6, and IL-1β in the respiratory system [4].

In addition to the inflammatory response, PM_2.5_ also increases free-radical production as it contains various organic compounds, such as lipopolysaccharides and polycyclic aromatic hydrocarbons [3]. Increased free-radical concentration leads to a reactive oxygen species (ROS)-mediated redox response, one of the main inducers of respiratory toxicity [5,6].

Increased ROS levels lead to pyroptosis, a type of programmed cell death mediated by the nuclear factor kappa-light-chain-enhancer of activated B cells (NF-κB) [7]. NF-κB increases the formation of the NLR family pyrin domain-containing 3 (NLRP3) inflammasome, which consists of NLRP3, pro-caspase-1, and inflammasome adaptor apoptosis-associated speck-like protein containing a c-terminal caspase recruitment domain (ASC) [8,9]. The NLRP3 inflammasome cleaves pro-caspase-1 into its activated form, caspase-1 [8,9]. Caspase-1 cleaves pro-IL-1β and pro-IL-18 into IL-1β and IL-18, respectively, aggravating inflammation [10,11,12]. Caspase-1 also cleaves Gasdermin D (GSDMD) into the N-terminal fragment of GSDMD (GSDMD-NT), which forms plasma pores [10,11] that release IL-1β and IL-18 into the extracellular space. This enhances inflammation, causing swelling of the cell and membrane rupture that results in pyroptosis [10,13,14,15,16]. Pyroptosis has been observed in respiratory cells exposed to PM_2.5_, such as human bronchial epithelial cells and A549 cells [17,18]. Similarly, PM_2.5_-treated mice showed increased pyroptosis in the respiratory system [19].

RAC family small GTPase 1 (RAC1) is involved in various signaling pathways related to inflammation, and inhibiting RAC1 leads to decreased inflammation during lung injury [20,21]. Administration of RAC1 inhibitor (NSC23766) decreased inflammation in PM_2.5_-exposed mice lungs [22]. RAC1 is also involved in the activation of the dinucleotide phosphate (NADPH) oxidase (NOX) complex, which increases ROS levels [23].

The NOX enzyme complex, such as NOX1 and NOX2, comprises membrane and cytosolic components, including RAC1 [24]. Structurally, NOX1, NOX2, NOX3, and NOX4 are made of p22^phox^ membrane subunits [25,26,27]. Functionally, NOX1, NOX2, and NOX4 are involved in the formation of NLRP3 inflammasome that increases ROS production [28,29].

Endogenous 8-hydroxydeoxyguanosine (8-OHdG) is generally considered a surrogate marker of ROS-induced oxidative damage to the DNA as it is a nucleoside with an oxidatively modified base [30]. Ironically, exogenously administered 8-OHdG exhibits an antioxidant effect by decreasing ROS generation by inhibiting RAC1 and NOX complex activation [30]. Exogenous 8-OHdG is also involved in decreasing the levels of inflammatory cytokines such as IL-6, IL-1β, and TNF-α by inhibiting NOX complex activation in the fat tissue or lipopolysaccharide-treated macrophages [31,32]. Moreover, exogenous 8-OHdG inhibits NF-κB activity and its downstream effects, such as NOX1 activation, in the gastrointestinal tract [33].

Therefore, we hypothesize that exogenous 8-OHdG inhibits the NOX complex in the PM_2.5_-treated cells by inhibiting RAC1 activation. Decreased NOX activity leads to decreased ROS synthesis and NLRP3 inflammasome formation, which eventually decreases inflammation in respiratory cells. Thus, we aimed to determine whether exogenous 8-OHdG could decrease inflammation, ROS generation, and pyroptosis in the PM_2.5_-treated human bronchial epithelial cell line BEAS-2B.

## 2. Materials and Methods

### 2.1. Reagents

Commercial PM_2.5_ (Diesel particulate matter; NIST^®^ SRM^®^ 1650b, Sigma-Aldrich, St. Louis, MO, USA) and tetrahydrochloride (NSC-23766; RAC1 inhibitor; N5412, Sigma-Aldrich) were purchased. The reagent 8-OHdG was donated by Myung-Hee Chung (Lee Gil Ya Cancer and Diabetes Institute, Gachon University, Republic of Korea). CellRox^®^ oxidative stress reagents (Sigma-Aldrich) were used in the intracellular oxidative stress detection experiment. We also purchased anti-NOX1 (PA5-39281, Invitrogen, Waltham, MA, USA), anti-NOX2 (ab129068, Abcam, Waltham, MA, USA), anti-NOX3 (ab81864, Abcam), anti-NOX4 (ab109225, Abcam), anti-RAC1 (05-389, Millipore, MA, USA), anti-p22^phox^ (#27967S, Cell Signaling Technology, Danvers, MA, USA), anti-NLRP3 (#15101, Cell Signaling Technology), anti-pro-caspase-1 (#3866, Cell Signaling Technology), anti-cleaved-caspase-1 (#4199T, Cell Signaling Technology), anti-precursor-IL-1β (#12703, Cell Signaling Technology), anti-mature-IL-1β (#83186T, Cell Signaling Technology), and anti-ASC (A96472, Antibodies.com, St Louis, MO, USA) antibodies. β-Actin (SC-47778, Santa Cruz, TX, USA) was used as an internal control for the Western blot analysis. The secondary antibodies HRP-labeled goat anti-rabbit IgG and HRP-labeled goat anti-mouse IgG were obtained from Cell Signaling Technology. Human IL-1β, IL-6, and IL-18 ELISA kits were purchased from R&D (Santa Clara, CA, USA).

### 2.2. Cell Culture and Treatment

Human bronchial epithelial cells (BEAS-2B) were obtained from the ATCC (Manassas, VA, USA). The cells were maintained in DK-SFM medium (GIBCO BRL, Gaithersburg, MD, USA) supplemented with 1% keratin (GIBCO BRL) and 1% penicillin–streptomycin solution (Saturius, Kibbutz Beit-Haemek, Israel) and incubated in a humidified atmosphere containing 5% CO_2_. PM_2.5_ was dissolved in 10% dimethyl sulfoxide, while 8-OHdG was dissolved in phosphate-buffered saline (PBS). For experiments involving PM_2.5_ exposure, 5 or 10 μg/mL 8-OHdG was used. The growing cells were supplemented with serum-free culture medium and continuously incubated at 37 °C for 24 h. The PM_2.5_ suspensions were always sonicated and vortexed before experiments.

### 2.3. Cell Viability Assay

Cell viability was measured using a Cell Counting Kit-8 (CCK-8 assay; DoJinDo Laboratories Co., Ltd., Kumamoto, Japan) and analyzed according to the manufacturer’s instructions. Cells were seeded in a 96-well microplate at a density of 1 × 10^4^ cells/well and treated with media containing various doses of PM_2.5_ and 8-OHdG at 37 °C for 24 h. The absorbance of the samples was determined at 450 nm using a microplate reader (SpectraMAX^®^ i3X, Molecular Devices, San Jose, CA, USA). The experiment was performed in triplicate.

### 2.4. Cell Toxicity Assay

BEAS-2B cells were seeded into a 96 well-plate (1 × 10^4^ cells/well) and exposed to PM_2.5_ or 8-OHdG depending on the treatment group. Damaged cells were quantified by measuring the amount of LDH released into the culture medium.

### 2.5. Intracellular ROS Production

CellRox^®^ oxidative stress reagents (Sigma-Aldrich) were used according to the manufacturer’s instructions to measure the intracellular ROS levels. BEAS-2B cells were seeded at a density of 2 × 10^4^ cells/well in an eight-well chamber slide and incubated for 24 h, followed by treatment with PM_2.5_ or 8-OHdG for another 24 h. After washing twice with PBS, 5 μM CellROX^®^ green reagent was added to the cells, which were incubated for 30 min at 37 °C away from light. DAPI was used to stain the nuclei. Next, the cells were incubated in 3.7% paraformaldehyde for 15 min at 37 °C to perform cell fixation. Observations were made using an inverted microscope (Flowview FV1000, Olympus, Tokyo, Japan), and intracellular fluorescence values (excitatio*n* = 485 nm; emissio*n* = 520 nm) were measured using Image J software (version 1.53t, 24 August 2022 (upgrade)).

### 2.6. Western Blotting Analysis

The cells were treated with or without PM_2.5_ and 8-OHdG for 24 h. After centrifugation at 12,000 rpm for 10 min, 10 μg of total protein from each group was subjected to electrophoresis on SDS-PAGE gels and transferred onto polyvinylidene fluoride membranes (Millipore). After incubation in a blocking buffer (5% skim milk in 1× PBS containing 0.1% Tween-20) for 1 h at 37 °C, the membranes were incubated overnight with the primary antibodies at 4 °C. The membranes were then rinsed with PBS-T and probed with the corresponding secondary antibodies for 1 h at 37 °C. The blots were visualized using an Immobilon Western Chemiluminescent HRP Substrate (EMD Millipore). Images of the Western blotting products were captured and analyzed using a ChemiDocTM Imaging System (Bio-Rad Laboratories, Inc., Hercules, CA, USA). Band density was measured using Image J software (version 1.53t, 24 August 2022 (upgrade)).

### 2.7. Detection of Active RAC1

An RAC1 pulldown assay was performed using a commercial kit (Cat No. 17-283, Millipore) according to the manufacturer’s instructions. Briefly, cells were washed with ice-cold PBS and lysed with lysis buffer containing freshly added 1× protease inhibitor cocktail (ZenDEPOT, TX, USA) on ice for 5 min. After centrifugation at 14,000× *g* for 15 min at 4 °C, the supernatant was mixed with glutathione agarose (Sigma-Aldrich) resins and incubated at 4 °C for 1 h. The resin was then washed three times with 1× cell lysis/binding/wash buffer. The resin-bound GTP–RAC1 was eluted with a 5× SDS sample buffer, followed by Western blotting analysis using mouse anti-human RAC1 antibodies.

### 2.8. ELISA

BEAS-2B cells seeded in six-well plates were separately treated with 10 μg/mL PM_2.5_ or 8-OHdG 5 for 24 h. The culture medium was then collected and stored at −80 °C until they were assayed for IL-1β, IL-6, and IL-18 using ELISA kits according to the manufacturer’s instructions. To further explore whether 8-OHdG decreases PM_2.5_-induced inflammatory cytokine levels, we concurrently exposed the cells to 80 μg/mL PM_2.5_ and 5 or 10 μg/mL 8-OHdG, and estimated the IL-1β, IL-6, and IL-8 levels in the medium. The absorbance was determined at 450 nm (excitation) and at 570 nm (emission) using a microplate reader (SpectraMAX^®^ i3X, Molecular Devices, San Jose, CA, USA).

### 2.9. Statistical Analysis

Student’s *t*-test was used to analyze the data. All statistical analyses were performed using SPSS software (SPSS 10.0 version, New York, NY, USA). Data are presented as the mean ± standard deviation. *p* < 0.05 was considered a statistically significant difference.

## 3. Results

### 3.1. PM_2.5_ Exposure Decreased BEAS-2B Cell Viability

**Figure 1:**
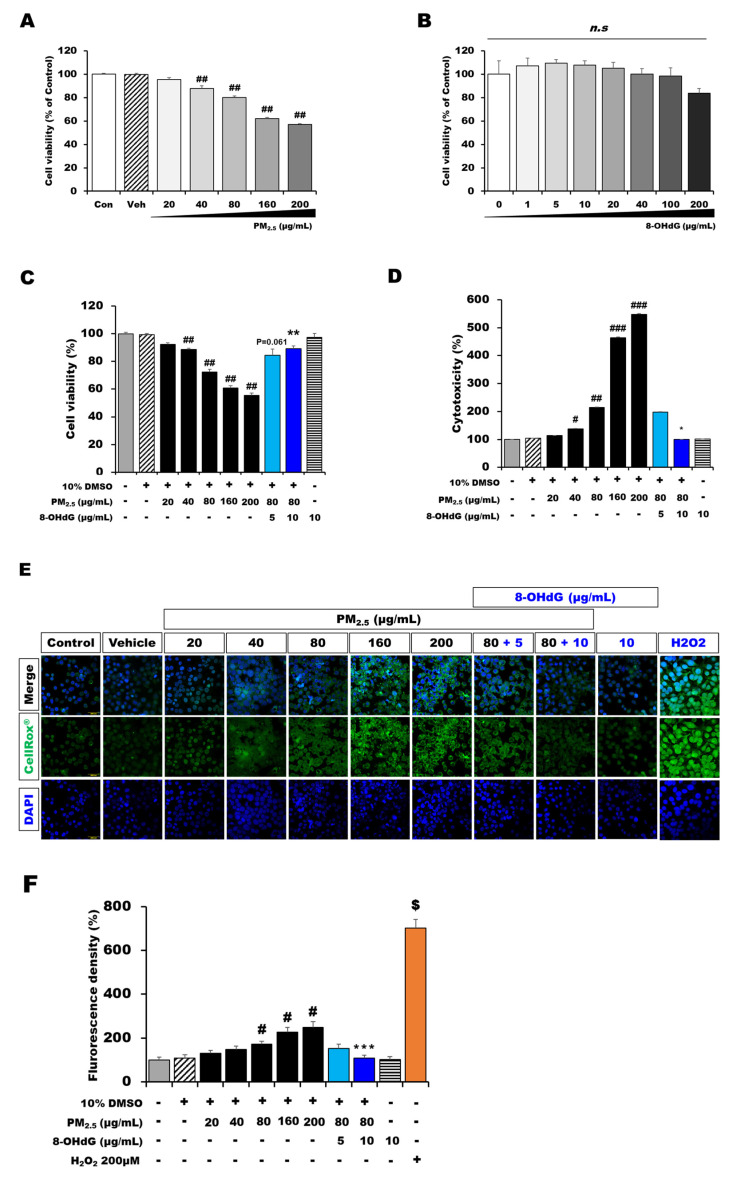
**Treatment with 8-OHdG increases cell viability in PM_2.5_-treated BEAS-2B cells.** Cells were treated with PM_2.5_ and/or 8-OHdG for 24 h at various concentrations. (**A**–**C**) Cell viability was measured using the MTT assay. (**D**) Cell cytotoxicity was measured using the lactate dehydrogenase assay. (**E**) The cells were stained with CellRox^®^ (green) to detect the cytoplasm and DAPI (blue) to detect the nucleus. (**F**) Quantification of the colocalization of intercellular ROS production using densitometric analysis. As a positive control, cells were treated with 200 μM H_2_O_2_ for 1 h. Scale bar, 200 μm. Results are shown as the mean ± SD of three independent experiments (*n* = 3). ^#^ *p* < 0.05, ^##^ *p* < 0.01, and ^###^ *p* < 0.001 versus the vehicle group; * *p* < 0.05, ** *p* < 0.01, and *** *p* < 0.001 versus the PM_2.5_-treated group; ^$^ *p* < 0.05 versus the control group. PM_2.5_, particulate matter 2.5; 8-OHdG, 8-hydroxydeoxyguanosine; ROS, reactive oxygen species.

The appropriate concentrations for the PM_2.5_ and 8-OHdG treatments were determined using the CCK-8 or lactate dehydrogenase (LDH) assays. BEAS-2B cell viability significantly decreased at PM_2.5_ concentrations higher than 40 μg/mL in the PM_2.5_-treated group (Figure 1A). The cell viability decreased by 20% and 38% in the groups treated with 80 μg/mL and 160 μg/mL PM_2.5_, respectively (*n* = 3, *p* < 0.001 vs. the control and vehicle groups). However, no significant difference in cell viability was observed between the control and treatment groups at 8-OHdG concentrations of 1–200 μg/mL (Figure 1B).

On the basis of these cell viability and cytotoxicity results, 80 μg/mL was chosen as the PM_2.5_ concentration for further in vitro experiments because this concentration elicited an inflammatory response and induced 20–30% cytotoxicity.

### 3.2. Exogenous 8-OHdG Attenuated PM_2.5_-Induced Cell Injury

CCK and LDH assays were performed to evaluate the effect of 8-OHdG treatment on cell viability and toxicity in PM_2.5_-treated BEAS-2B cells. As shown in Figure 1C,D, cell viability decreased to 28% after treatment with 80 μg/mL PM_2.5_ and was notably recovered by 15% following 8-OHdG treatment. Similarly, PM_2.5_-induced cytotoxicity was notably inhibited by 46% following treatment with 8-OHdG (Figure 1D). Furthermore, the attenuating effect of 8-OHdG was higher at 10 μg/mL than at 5 μg/mL. Hence, further experiments were performed to evaluate the efficacy of 8-OHdG in decreasing NLRP3 inflammasome activation and pyroptosis induction via RAC1 inhibition at a concentration of 10 μg/mL.

### 3.3. Exogenous 8-OHdG Treatment Decreased ROS Production in PM_2.5_-Treated BEAS-2B Cells

Intracellular ROS levels were measured using a CellRox^®^ oxidative stress assay to determine whether 8-OHdG reduced PM_2.5_-induced ROS production. BEAS-2B cells exposed to PM_2.5_ displayed brighter green fluorescence than the control. The cells simultaneously pretreated with 8-OHdG and PM_2.5_ displayed light-green fluorescence similar to the control (Figure 1E). We quantified the ROS levels by measuring the fluorescence intensity and confirmed that intracellular ROS levels were elevated in BEAS-2B cells exposed to 80 μg/mL PM_2.5_ but significantly decreased after incubation with 8-OHdG (Figure 1F).

### 3.4. Exogenous 8-OHdG Attenuated PM_2.5_-Induced Inflammatory Cytokine Secretion

**Figure 2:**
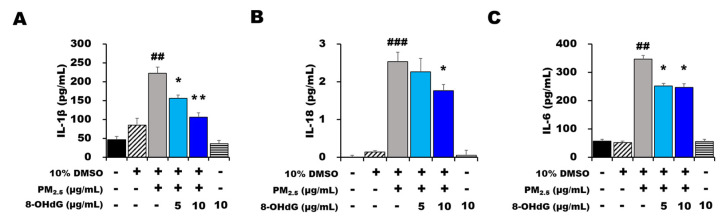
**Exogenous 8-OHdG administration inhibits inflammatory cytokine production in PM_2.5_-induced BEAS-2B cells.** BEAS-2B cells were treated with PM_2.5_ (80 μg/mL) and/or 8-OHdG (5 or 10 μg/mL) for 24 h, and cytokine secretion was evaluated. (**A**) IL-1β, (**B**) IL-18, and (**C**) IL-6 concentrations were measured using enzyme-linked immunosorbent assay kits. Results are shown as the mean ± SD of three independent experiments (*n* = 3). ^##^ *p* < 0.01, and ^###^ *p* < 0.001 versus the vehicle group; * *p* < 0.05, and ** *p* < 0.01 versus the PM_2.5_-treated group. PM_2.5_, particulate matter 2.5; 8-OHdG, 8-hydroxydeoxyguanosine; IL-1β, interleukin-1β; IL-6, interleukin-6; IL-18, interleukin-18.

To quantify the levels of inflammatory cytokines released by BEAS-2B cells, we analyzed IL-1β, IL-6, and IL-8 expression using enzyme-linked immunosorbent assay (ELISA) kits. As expected, PM_2.5_ treatment showed significantly increased expression levels of the inflammatory cytokines IL-1β, IL-6, and IL-8 in a concentration-dependent manner compared with the control and vehicle-treated groups (Figure 2).

Simultaneous exposure of the cells to PM_2.5_ and 8-OHdG showed that pretreatment significantly reduced IL-1β, IL-6, and IL-18 expression (Figure 2). These results prove that 8-OHdG pretreatment recovered cell viability, mitigated cytotoxicity, and reduced ROS production and PM_2.5_-induced inflammatory cytokine secretion.

### 3.5. Exogenous 8-OHdG Alleviated NLRP3 Inflammasome Activation in PM_2.5_-Treated BEAS-2B Cells

**Figure 3:**
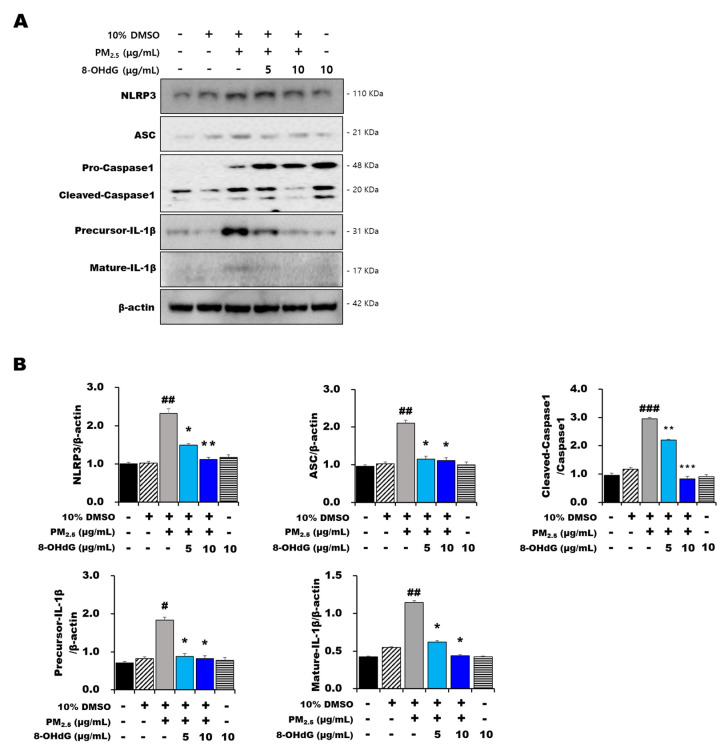
**Exogenous 8-OHdG inhibits inflammatory responses in PM_2.5_-induced BEAS-2B cells.** BEAS-2B cells were treated with PM_2.5_ (80 μg/mL) and/or 8-OHdG (5 or 10 μg/mL) for 24 h. (**A**,**B**) Western blotting analysis showing NLRP3, ASC, caspase-1, and IL-1β expression. Quantification of protein expression was performed using the band intensities and normalized against that of β-actin. A representative blot is shown from three independent experiments (*n* = 3). Results are shown as the mean ± SD of three independent experiments (*n* = 3). ^#^ *p* < 0.05, ^##^ *p* < 0.01, and ^###^ *p* < 0.001 versus the vehicle group; * *p* < 0.05, ** *p* < 0.01, and *** *p* < 0.001 versus the PM_2.5_-treated group. PM_2.5_, particulate matter 2.5; 8-OHdG, 8-hydroxydeoxyguanosine; NLRP, NLR family pyrin domain-containing; ASC, apoptosis-associated speck-like protein containing a CARD c-terminal caspase recruitment domain; IL-1β, interleukin-1β.

To further assess the role of PM_2.5_ in epithelial cell inflammation, we performed Western blotting to detect NLRP3 inflammasome activation. The expression of NLRP3, ASC, cleaved caspase-1, precursor IL-1β, and mature IL-1β significantly increased in PM_2.5_-treated BEAS-2B cells compared with that in the control group (Figure 3A,B). However, the expression of inflammasome-related proteins significantly decreased after preincubation with 8-OHdG. These results indicated that 8-OHdG attenuates the NLRP3/caspase-1 pathway activated by PM_2.5_ treatment.

### 3.6. Exogenous 8-OHdG Inhibits NOX1 Expression and RAC1 Activation in PM_2.5_-Treated BEAS-2B Cells

**Figure 4:**
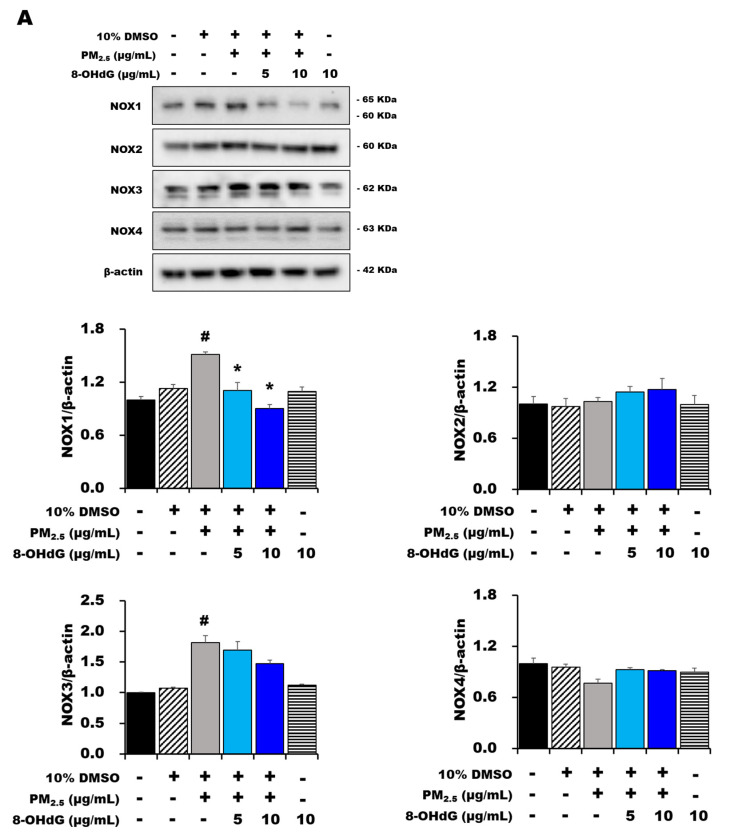

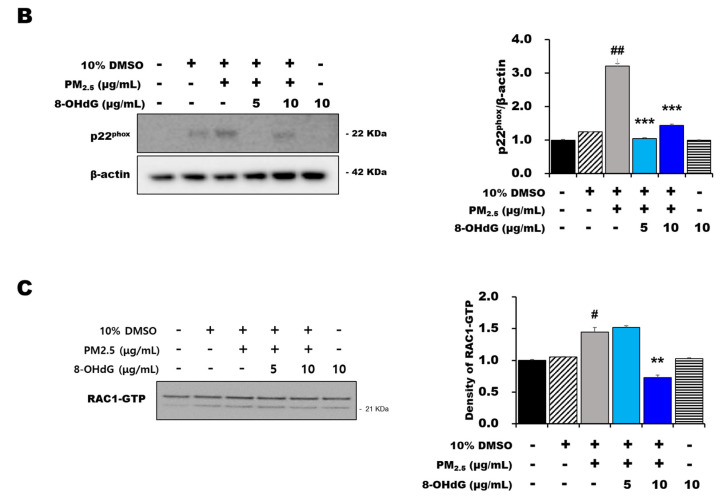
**Exogenous 8-OHdG inhibits NOX signaling in PM_2.5_-induced BEAS-2B cells.** BEAS-2B cells were treated with PM_2.5_ (80 μg/mL) and/or 8-OHdG (5 or 10 μg/mL) for 24 h. Western blot analysis showing the expression of (**A**) NOX1–4, (**B**) p22^phox^, and (**C**) RAC1–guanosine triphosphate (GTP) activity. Quantification of protein expression was performed using the band intensities and was normalized against that of β-actin. A representative blot is shown from three independent experiments (*n* = 3). The results are shown as the mean ± SD of three independent experiments (*n* = 3). ^#^ *p* < 0.05, and ^##^ *p* < 0.01 versus the vehicle group; * *p* < 0.05, ** *p* < 0.01, and *** *p* < 0.001 versus the PM_2.5_-treated group. PM_2.5_, particulate matter 2.5; 8-OHdG, 8-hydroxydeoxyguanosine.

Previous studies state that exogenous 8-OHdG alleviates liver fibrosis and epithelial–mesenchymal transition by inhibiting NOX-derived ROS formation [34]. Therefore, we determined the protein expression of NOX1, NOX2, NOX3, and NOX4 using immunoblotting and found that only NOX1 expression was significantly elevated in BEAS-2B cells exposed to PM_2.5_ compared with that of the control group. However, pretreatment with 8-OHdG inhibited NOX1 expression in PM_2.5_-treated cells (Figure 4A).

The NADPH oxidase NOX1 consists of the transmembrane proteins NOX1 and p22^phox^ and the cytosolic proteins NOXO1, NOXA1, and RAC1 [24]. Here, we additionally investigated p22^phox^ expression and RAC1 activation. Figure 4B,C show that the PM_2.5_-induced increase in p22^phox^ expression and RAC1 activation were significantly reversed after treatment with 8-OHdG. These results show that 8-OHdG reduces ROS generation by modulating NOX1/RAC1 activation.

### 3.7. RAC1 Inhibitor Downregulates NOX1/p22^phox^ Expression in PM_2.5_-Treated BEAS-2B Cells

NSC23766 is a specific RAC1 inhibitor that can be used to confirm the effect of 8-OHdG on RAC1-mediated NOX1/p22^phox^ expression and NLRP3 signaling. After treatment for 24 h with the RAC1 inhibitor, cell viability was measured using the CCK assay. NSC23766 had no effect on cell viability at concentrations of 0.1–10 μM (Figure S1). We preincubated the cells with NSC23766 and 8-OHdG and subsequently exposed them to PM_2.5_.

**Figure 5:**
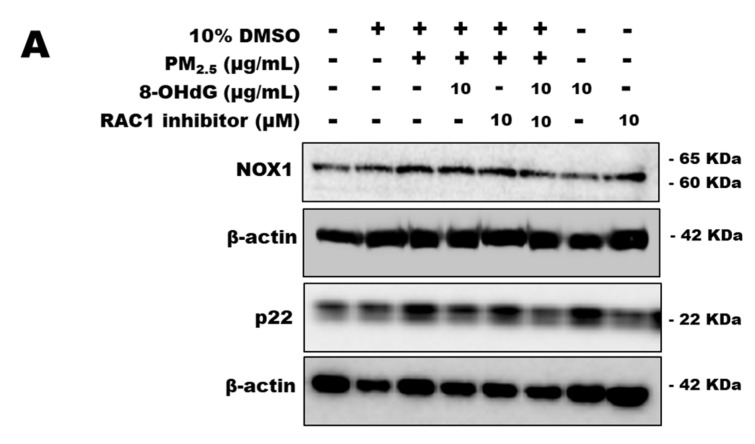

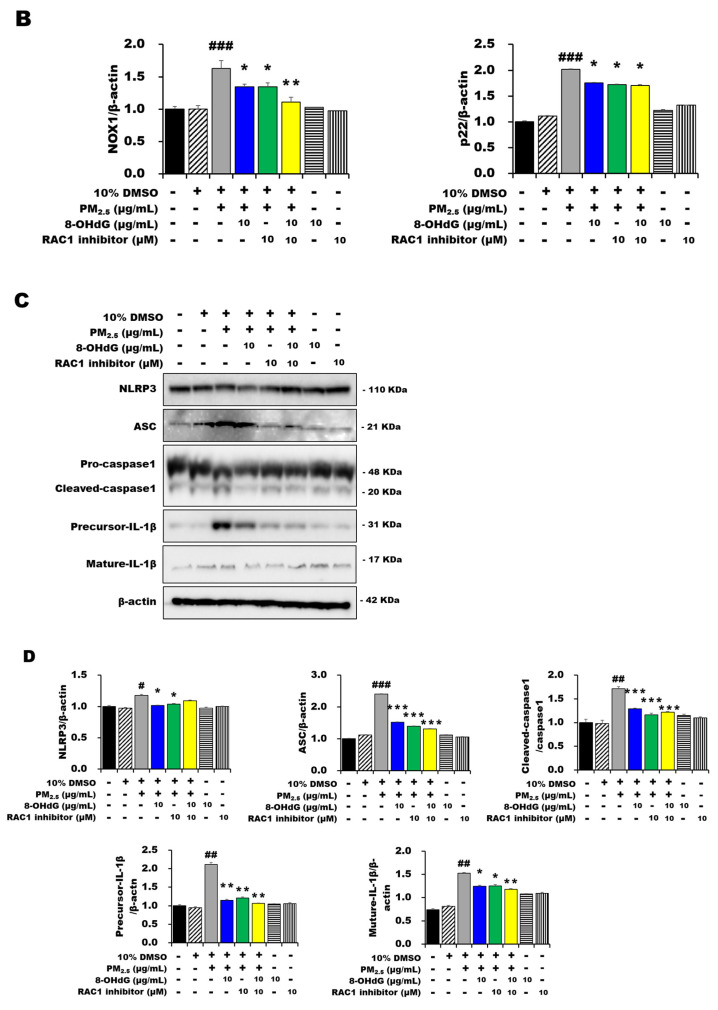
**The RAC1 inhibitor NSC23766 downregulates NOX1/p22^phox^ signaling and inhibits inflammatory responses in PM_2.5_-induced BEAS-2B cells.** BEAS-2B cells were treated with PM_2.5_ (80 μg/mL) or 8-OHdG (10 μg/mL) and RAC1 inhibitor (10 μM) for 24 h. Western blotting analysis showing NOX1, p22^phox^ (**A**,**B**), NLRP3, ASC, caspase-1, and IL-1β (**C**,**D**). Quantification of protein expression was performed using the band intensities and normalized against that of β-actin. A representative blot is shown from three independent experiments (*n* = 3). The results are shown as the mean ± SD of three independent experiments (*n* = 3). ^#^ *p* < 0.05, ^##^ *p* < 0.01, and ^###^ *p* < 0.001 versus the vehicle group; * *p* < 0.05, ** *p* < 0.01, and *** *p* < 0.001 versus the PM_2.5_-treated group. PM_2.5_, particulate matter 2.5; 8-OHdG, 8-hydroxydeoxyguanosine; NLRP, NLR family pyrin domain containing; ASC, apoptosis-associated speck-like protein containing a CARD c-terminal caspase recruitment domain.

Western blotting showed that NSC23766 significantly reduced NOX1 and p22^phox^ expression in cells exposed to PM_2.5_ (Figure 5A). Similarly, pretreatment with 8-OHdG inhibited NOX1/p22^phox^ expression. NOX1 expression was further reduced in cells simultaneously exposed to the RAC1 inhibitor and 8-OHdG (Figure 5A,B).

### 3.8. RAC1 Inhibitor Attenuates NLRP3 Inflammasome and Inflammatory Responses in PM_2.5_-Treated BEAS-2B Cells

Western blotting showed a significantly higher expression of NLRP3, ASC, cleaved caspase-1, precursor IL-1β, and mature IL-1β proteins in BEAS-2B cells treated with 80 μg/mL PM_2.5_ than in the control and vehicle groups (Figure 5C,D), similar to previous results. However, the expression of these proteins was reduced in PM_2.5_-treated cells after preincubation with an RAC1 inhibitor or 8-OHdG.

## 4. Discussion

In this study, we observed that 8-OHdG reduced the secretion of PM_2.5_-induced inflammatory cytokines, such as IL-1β and IL-18, in respiratory cells by decreasing NLRP3 inflammasome formation. Moreover, 8-OHdG decreased NLRP3 inflammasome activation by inhibiting RAC1 and NOX activity in BEAS-2B cells. RAC1 is associated with increased respiratory inflammation induced by PM_2.5_ [22]. PM_2.5_ also increases ROS generation [35], and RAC1 increases ROS levels by activating NOX proteins [23]. NOX1 comprises cytosolic components, such as NOX organizer 1 (a homolog of p47phox) and NOX activator 1 (a homolog of p67phox) [23,36,37]. The membrane subunit p22^phox^ and cytosolic subunits of Rac GTPase are required for NOX1 activation [23].

Increased ROS concentration induces NLRP3 inflammasome activation [38,39], and PM_2.5_-induced oxidative stress leads to NLRP3 inflammasome activation. This was reportedly inhibited by antioxidants such as *N*-acetyl-L-cysteine [40,41]. PM_2.5_ has been shown to increase NLRP3 inflammasome activity in the lungs, which increases the expression of caspase-1, IL-1β, and IL-18 and induces pyroptosis [42]. NLRP3-induced pyroptosis is exhibited in various clinical situations, such as acute lung injury, pulmonary fibrosis, and lung cancer [43,44,45,46,47,48,49,50].

In our study, PM_2.5_ treatment increased the expression of NLRP3, ASC, and cleaved caspase-1 and the levels of pro-IL-1β and mature IL-1β in BEAS-2B cells. Similarly, IL-1β and IL-18 levels were elevated in PM_2.5_-treated BEAS-2B cells. However, treatment with 8-OHdG attenuated these effects. Furthermore, the mRNA expression of NOX1, inflammation-related NLRP3 signaling molecules, and inflammatory cytokines was increased in BEAS-2B cells upon exposure to PM_2.5_. Conversely, their expression decreased after treatment with 8-OHdG (Supplementary Figure S2 and Supplementary Table S1). 

RAC1 functions as a molecular switch by binding with either guanosine diphosphate (GDP) or guanosine triphosphate (GTP) [51]. Binding with GTP activates RAC1; however, binding with GDP inactivates RAC1 [51]. The regulation of the binding of GDP or GTP to RAC1 is modulated by guanine nucleotide exchange factors (GEFs), GTPase-activating proteins (GAPs), and Rho guanine nucleotide dissociation inhibitors (RhoGDIs) [52,53,54]. GEFs remove GDP from RAC1, eventually offering free cellular GTP to bind with RAC1 [55,56].

The molecule 8-OHdG is known to exert inhibitory effects on RAC1 by binding with the RAC1–GEF complex [57]. This study demonstrated a reduction in the PM_2.5_-induced increase in RAC1 activity and NOX1 expression following treatment with 10 μg/mL 8-OHdG. Given that RAC1 activation is required to activate NOX1 and NOX2 [27], 8-OHdG seems to decrease NOX1 activity by inhibiting RAC1.

NSC23766 specifically prevents the conversion of RAC1–GDP to RAC1–GTP by competitively blocking the binding loop of RAC1-specific GEF [58]. Adding an RAC1 inhibitor to PM_2.5_-treated BEAS-2B cells decreased the expression of NOX1 and p22^phox^. Interestingly, the degree to which 8-OHdG attenuated the expression of these proteins was similar to that observed for the RAC1 inhibitor. NLRP3 inflammasome activation, which was evaluated on the basis of NLRP3, ASC, and cleave caspase-1 expression, was also decreased by both 8-OhdG and the RAC1 inhibitor to similar degrees. The expression of pro-IL-1β and IL-1β in PM_2.5_-treated BEAS-2B cells was also decreased by both 8-OhdG and the RAC1 inhibitor.

IL-1β is a pluripotent proinflammatory cytokine involved in various inflammation-related lung diseases [59]. Moreover, IL-1β leads to PM_2.5_-induced pulmonary inflammation [60]. IL-1β triggers leukocytes to secrete more cytokines, such as IL-6, and increases the migration of leukocytes, which enhances inflammation [61]. IL-1β is also involved in increasing IL-18 expression, which promotes the T-cell-induced immune response in airway inflammation [62,63]. IL-6 is an essential proinflammatory cytokine, the levels of which increase during acute inflammation due to environmental insult to the lungs [62,63].

In this study, we also observed increased IL-1β and IL-18 levels and IL-6 secretion in BEAS-2B cells following exposure to PM_2.5_, which decreased following treatment with 8-OHdG.

Reportedly, the average life span is shortened by 8.6 months by PM_2.5_ [64], which constitutes approximately 70% of inhalable particles in humans [65]. Therefore, increased daily exposure to PM_2.5_ by 10 µg/m^3^ increases the incidence of respiratory diseases by 2.07% [66,67]. As PM_2.5_ exerts a tremendous impact on the respiratory tract by increasing ROS concentration or inflammation that results in lung injury, various modalities to decrease ROS levels or inflammation are being widely evaluated. Exogenous 8-OHdG, which decreases ROS levels and attenuates inflammation by inhibiting RAC1 and NOXs, could be a viable candidate for decreasing PM_2.5_-induced lung injury. Nevertheless, this study had limitations. First, a broader spectrum of concentrations must be shown since 8-OHdG is known to exert divergent effects due to its oxidative potential. Our experiments were performed using only two doses, 5 and 10 µg/mL. We focused on verifying whether low concentrations of 8-OHdG reduce the oxidative stress induced by PM_2.5_. Thus, although treatment with 8-OHdG alone did not affect cell viability, it was difficult to observe its effect on other categories of markers, such as inflammation. Further studies will be needed to explain the effect of different concentrations of 8-OHdG on these parameters. 

Second, although the ATCC recommended that BEAS-2B cells be cultured in BEGM, other media could be used for BEAS-2B culture. According to Zhao and Klimecki, fetal bovine serum showed cytotoxicity in BEAS-2B cells and decreased the expression of E-cadherin, an epithelial cell phenotype marker [68]. When cells lose their original characteristics, they exhibit various responses to stimuli, usually at varying degrees. We were not able to further examine the difference between the culture conditions and PM_2.5_ concentrations in BEAS-2B cells, which could have provided more information to better understand the degree of NOX expression after PM_2.5_ exposure. Therefore, it is necessary to characterize the cells more comprehensively according to the composition of the culture medium. 

Research to address these limitations will continue in the future. In this study, we found that exogenous 8-OHdG, which reduces ROS levels and attenuates inflammation by inhibiting RAC1 and NOX, can reduce lung damage caused by PM_2.5_ exposure and suggested possible candidates.

## 5. Conclusions

Our study showed that exogenous 8-OHdG decreased PM_2.5_-induced ROS generation and NLRP3 inflammasome formation, which was accompanied by reduced IL-6, IL-1β, and IL-18 levels in respiratory cells. The possible mechanism underlying these effects of 8-OHdG could be the inhibition of RAC1 and NOX1.

## Data Availability

All data generated or analyzed during this study are included in this published article and the Supplementary Materials.

## Supplementary Materials

The following supporting information can be downloaded at https://www.mdpi.com/article/10.3390/antiox12061189/s1: Figure S1. RAC1 inhibitor decreased dose-dependently cell viability in BEAS-2B cells; Figure S2. Treatment with 8-OHdG decreased PM_2.5_-induced mRNA expression of target genes in BEAS-2B cells; Table S1. qRT-PCR primer sequence information.
