# Supplementary material for: Exogenous 8-Hydroxydeoxyguanosine Attenuates PM2.5-Induced Inflammation in Human Bronchial Epithelial Cells by Decreasing NLRP3 Inflammasome Activation
Source: Antioxidants (Basel). 2023 May 31;12(6):1189. doi: 10.3390/antiox12061189 (PMC10295443; doi:10.3390/antiox12061189)
Supplement: Supplementary file 1 [file antioxidants-12-01189-s001.zip › antioxidants-2357144-supplementary.pdf]

antioxidants

MDPI

Figure S1. RAC1 inhibitor decreased dose-dependently cell viability in BEAS-2B cells. The results are shown as the mean ± SD: * $p < 0.05$, ** $p < 0.01$, and *** $p < 0.001$

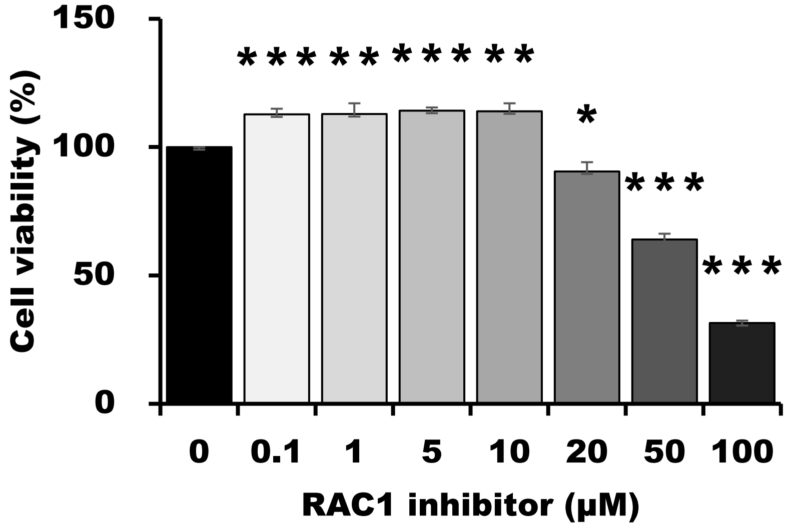

Figure S2. Treatment with 8-OHdG decreased PM2.5-induced mRNA expression of target genes in BEAS-2B cells.

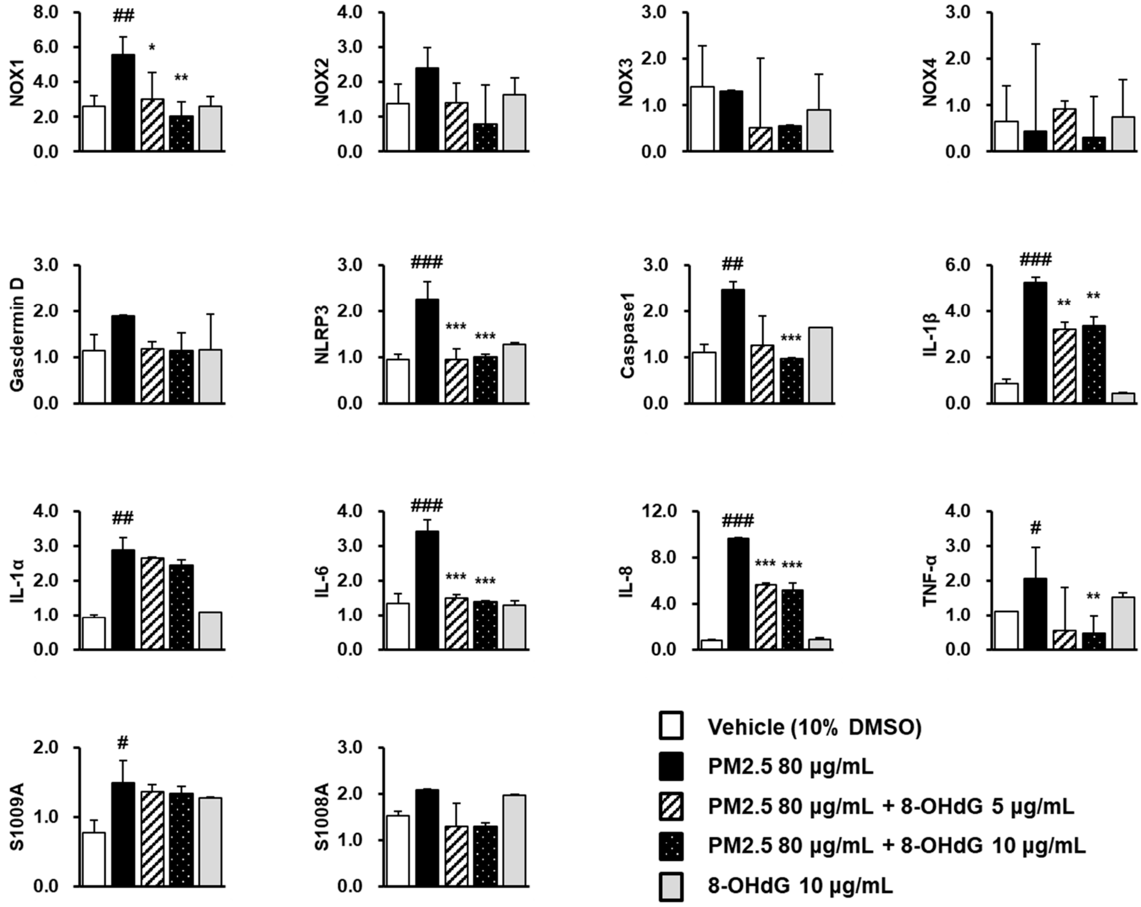

Table S1. qRT-PCR primer sequence information.

Target gene	Primer sequence
NOX1	F : 5′-AGGGCTTTCGAACAATA -3′<br>R : 5′-CCAGCACAGCTTCATAC-3′
NOX2	F : 5′-AACTGCTGGAGAGCCAGATG -3′<br>R : 5′-GCAAAGTGATTGGCCTGAGA-3′
NOX3	F : 5′-GCTATGCAGAATGGCAGACA -3′<br>R : 5′-TACAAGACCACAGGGCCTAA -3′
NOX4	F : 5′-GAGCCCAGATTCCAAGCTAATT -3′<br>R : 5′-GGCACAAAGGTCCAGAAATCC -3′
Gasdermin D1	F : 5′-GCCAGAAGAAGACGGTCACCATC-3′<br>R : 5′-TTCGCTCGTGGAACGCTTGTG-3′
NLRP3	F : 5′-GATCTTCGCTGCGATCAACA-3′<br>R : 5′-GGGATTCGAACACGTGCATTA-3′
Caspase1	F : 5′-AAGACCCGAGCTTTGATTGACTC -3′<br>R : 5′-AAATCTCTGCCGACTTTTGTTTCC -3′
IL-1β	F : 5′-CTGTCCTGCGTGTTGAAAGA-3′<br>R : 5′-TTCTGCTTGAGAGGTGCTCA-3′
IL-1α	F : 5′-ATCAGTACCTCACGGCTGT-3′<br>R : 5′-TGGGTATCTCAGGCATCTCC-3′
IL-6	F : 5′-CCAGAACAGATTTGAGAG-3′<br>R : 5′-CTACATTTGCCGAAGAGC-3′
IL-8	F : 5′-GCGCAGTTTTGCCAAGGAGT-3′<br>R : 5′-CTCTGCACCCAGTTTTCCTT-3′
TNF-α	F : 5′-CCCTCACACTCAGATCATCTTCT-3′<br>R : 5′-GCTACGACGTGGGCTACAG-3′
S1009A	F : 5′-TGGAGGACCTGGACACAAATG-3′<br>R : 5′-TCGTCACCCTCGTGCATCTT-3′
S1008A	F : 5′-GCTAGAGACCGAGTGTCCTCAG-3′<br>R : 5′-GCCCATCTTTATCACCAGAATG-3′
GAPDH	F : 5′-TGCACCACCAACTGCTTAGC-3′<br>R : 5′-GGCATGGACTGTGGTCATGAG-3′
